# Supplementary material for: Depth-resolved fiber photometry of amyloid plaque signals in freely behaving Alzheimer’s disease mice
Source: Neurophotonics. 2025 Sep 23;12(3):035014. doi: 10.1117/1.NPh.12.3.035014 (PMC12456868; doi:10.1117/1.NPh.12.3.035014)
Supplement: Supplementary file 1 [file NPh_012_035014_SD001.pdf]

Depth-resolved fiber photometry of amyloid plaque signals in freely behaving Alzheimer's disease mice

Nicole Byron, Niall McAlinden, Filippo Pisano, Marco Pisanello, Jacques Ferreira, Cinzia Montinaro, Keith Mathieson, Massimo De Vittorio, Ferruccio Pisanello, Shuzo Sakata

Supplemental Table 1. Information about the mice used throughout this study. The experiment each mouse was used for is listed as flat fiber (FF), *ex vivo* (ExV) or tapered fiber (TF). Mice that were excluded and the appropriate reason is noted.

ID	Genotype	Sex	Age @ experiment start (mo)	Excluded?	Reason	Experiment type	Figure no.
1	+	F	6.9			FF	1
2	+	M	8.5			FF	1
3	+	M	8.4			FF	1
4	+	M	6.8	Y	incomplete recording	FF	
5	+	M	7.6			FF	1
6	+	M	6.9			FF	1
7	+	M	7.1			FF	1
8	-	F	8.0			FF	1
9	-	F	6.9			FF	1
10	-	F	8.4			FF	1
11	-	M	8.4			FF	1
12	-	M	6.9	Y	incomplete recording	FF	
13	-	M	7.0			FF	1
14	-	M	7.1			FF	1
15	-	M	7.1			FF	1
16	+	M	9.1			ExV	2
17	+	M	9.1			ExV	2
18	-	M	9.1			ExV	2
19	+	F	4.3	Y	incorrect laser power estimation	TF	
20	+	F	4.8			TF	3
21	+	F	6.0			TF	3
22	+	F	3.0			TF	3
23	+	F	3.3			TF	3
24	+	F	8.0	Y	histology registration error	TF	
25	+	F	3.5			TF	3
26	+	F	6.8			TF	3
27	-	F	4.6	Y	incorrect laser power estimation	TF	
28	-	F	4.9			TF	3
29	-	F	6.0			TF	3
30	-	F	2.9			TF	3
31	-	F	3.3			TF	3
32	-	F	8.0			TF	3
33	-	F	3.5			TF	3
34	+	M	4.9			TF	3
35	+	M	6.0			TF	3
36	+	M	3.0			TF	3
37	+	M	3.9			TF	3
38	+	M	3.6			TF	3
39	+	M	6.8			TF	3
40	-	M	4.7	Y	incorrect laser power estimation	TF	
41	-	M	3.0			TF	3
42	-	M	3.9	Y	histology registration error	TF	
43	-	M	5.7			TF	3

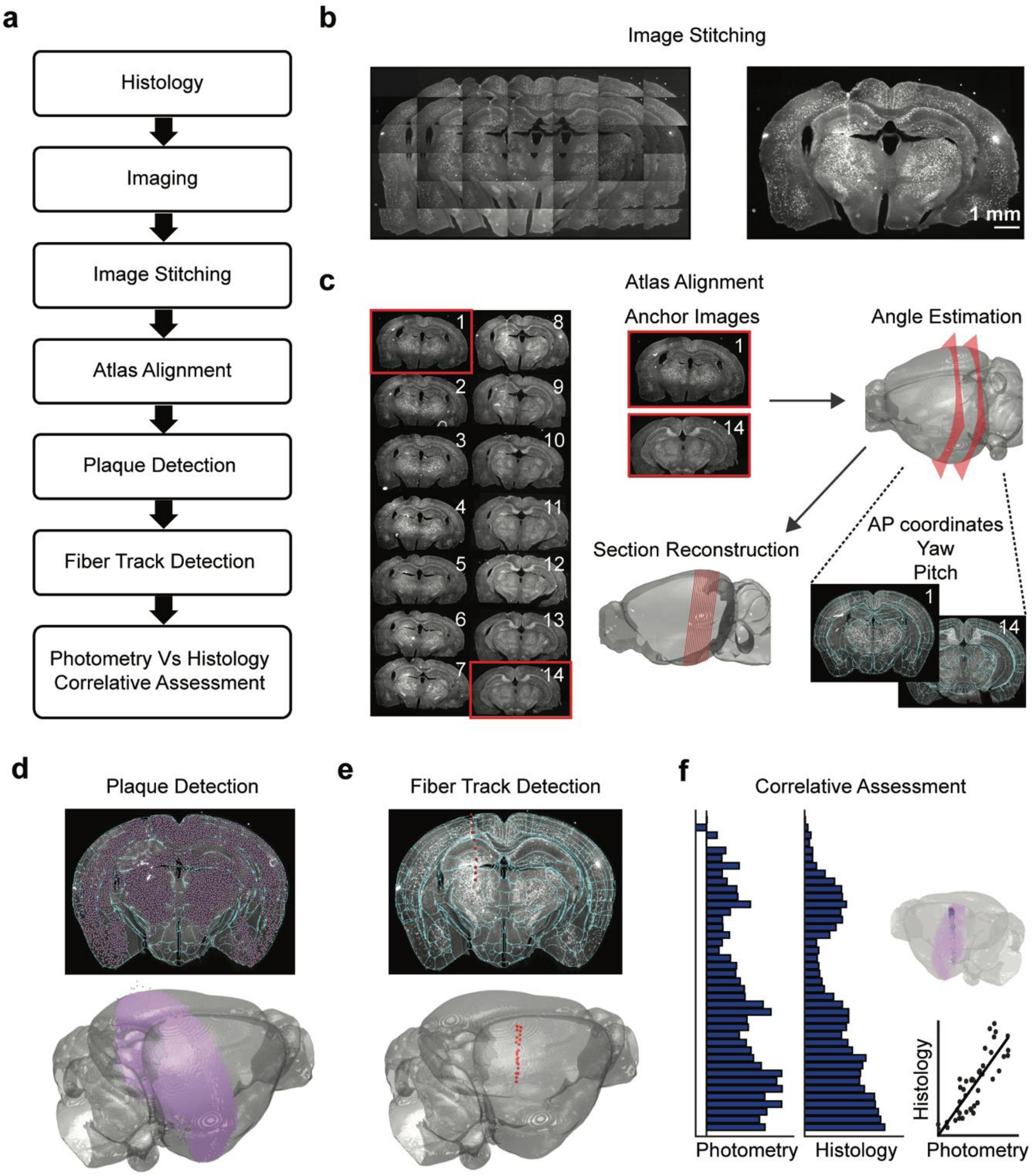

Supplemental Figure 1. Protocol for reconstruction, registration and quantification of Methoxy-X04 stained histological sections. (**a**) Flow chart of the protocol. (**b**) Image stitching of all original images (*left*) to the final stitched image (*right*). Scale bar: 1 mm. (**c**) The first and final stitched image (*shown with red border*) are anchor images used for atlas alignment. Anchor images are used to determine several alignment parameters before all image slices are reconstructed onto the atlas. (**d**) *Top*, Automated plaque detection on an aligned brain slice. *Bottom,* Detected plaques on all aligned brain slices, visualized on a whole brain. Purple signals represent a detected plaque. (**e**) *Top*, Manual fiber track detection on an aligned brain slice. *Bottom,* Detected fiber track on all aligned brain slices, visualized on a

whole brain. Red signals represent the manually annotated fiber track. (**f**) Plaques within 250-µm of the manually annotated fiber track (*red*), on the contralateral hemisphere are quantified. Blue signals represent quantified plaques. Correlation analysis was completed on photometry and histology depth profiles.

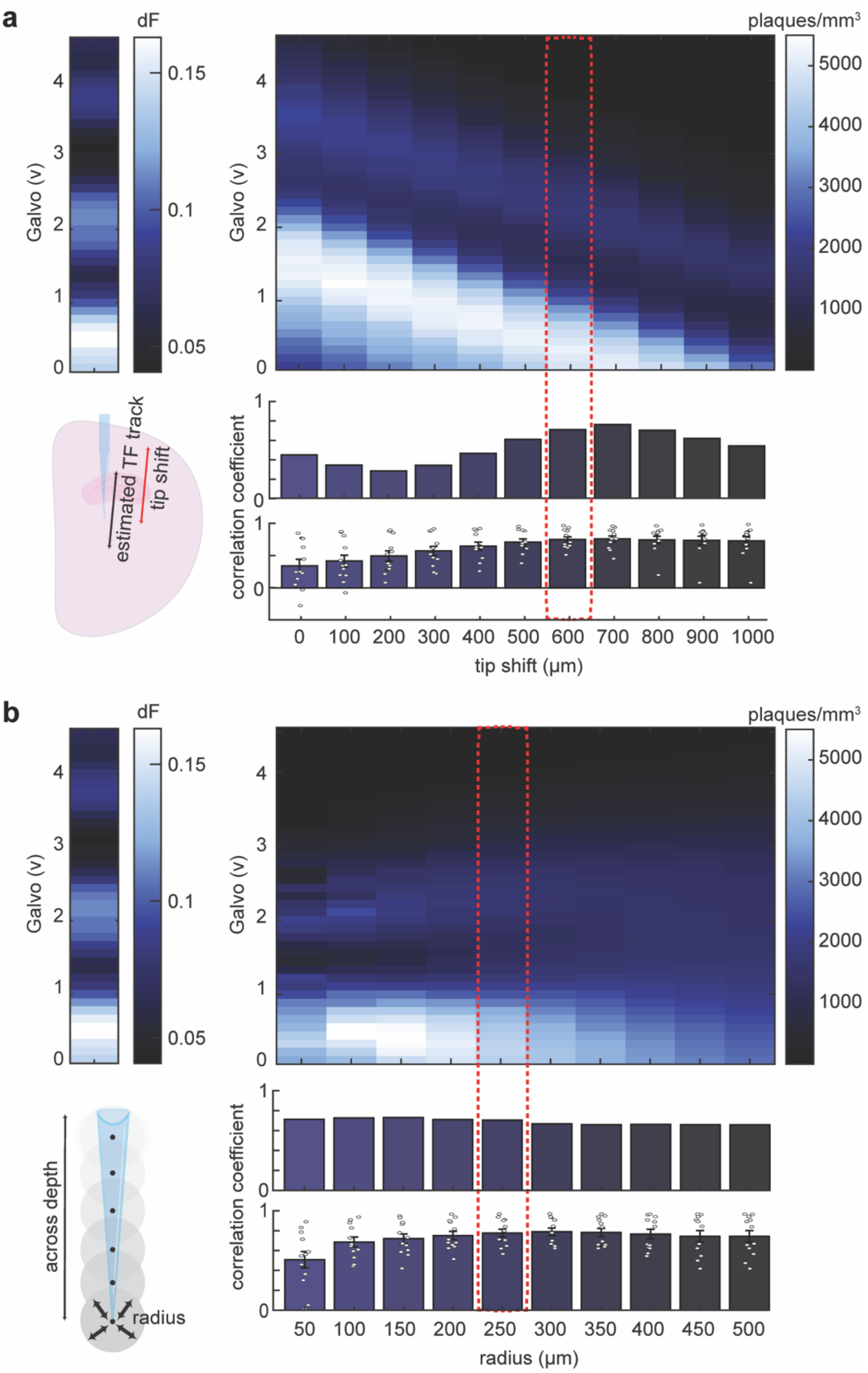

Supplemental Figure 2. Systemic assessment of the histological quantification parameters. (**a**) *Bottom left,* TFs are minimally invasive which makes it challenging to accurately identify the TF tip on histological sections. Often, this led to an error in the estimated penetration track for TF implants. This error could be corrected by shifting the TF penetration track up several hundred micrometers. *Top left,* Heatmap of the mean fluorescent signals from 210-240 minutes on day 1 of the TF recording. *Top right*, Heatmap of the histology depth

profiles with TF tip shifts from 0 to 1000 μm. *Bottom right*, Top panel shows the correlation coefficient when comparing the photometry and histology depth profiles for this example. Bottom panel shows the correlation coefficient across all mice. Red dashed line shows a tip shift of 600 μm provides the best correction. (**b**) *Bottom left,* The radius of the sphere represents the distance threshold for quantified plaques used at each galvo measure. *Top left,* Heatmap of the mean fluorescent signals from 210-240 minutes on day 1 of the TF recording. *Top right*, Heatmap of the histology depth profiles with radius from 50 to 500 μm. *Bottom right*, Top panel shows the correlation coefficient when comparing the photometry and histology depth profiles for this example. Bottom panel shows the correlation coefficient across all mice. Red dashed line shows a radius of 250 μm provides the best reflection of light propagation from the TF.

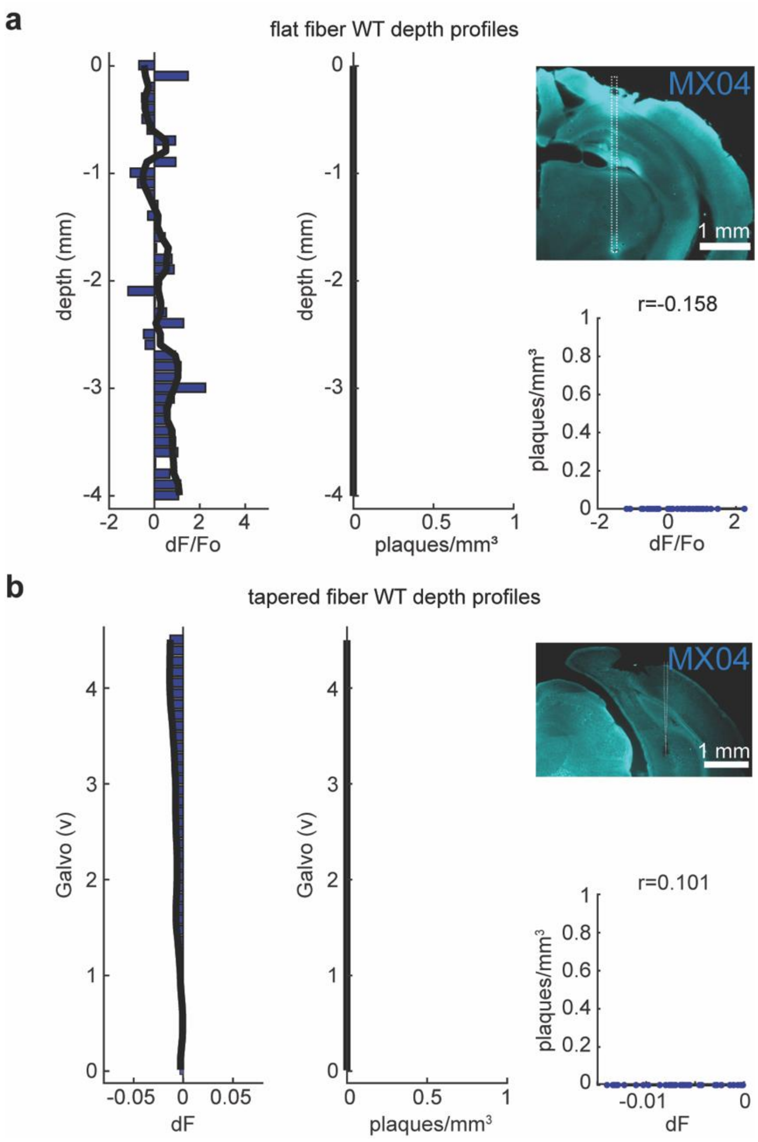

Supplemental Figure 3. Fiber photometry and histological assessment of Methoxy-X04 signals in WT mice. Examples of flat fiber-based (**a**) and TF-based (**b**) experiments. *Left,* Example *in vivo* fluorescent and post-mortem histology depth profiles. Solid line shows the median smoothed signal (window size: 4). *Top right*, Coronal brain slice showing the fiber penetration track (white dashed line). Scale: 1 mm. *Bottom right*, Correlation analysis comparing photometry and histology depth profiles (Spearman's Rho correlation test). Black line shows a fitted linear regression.

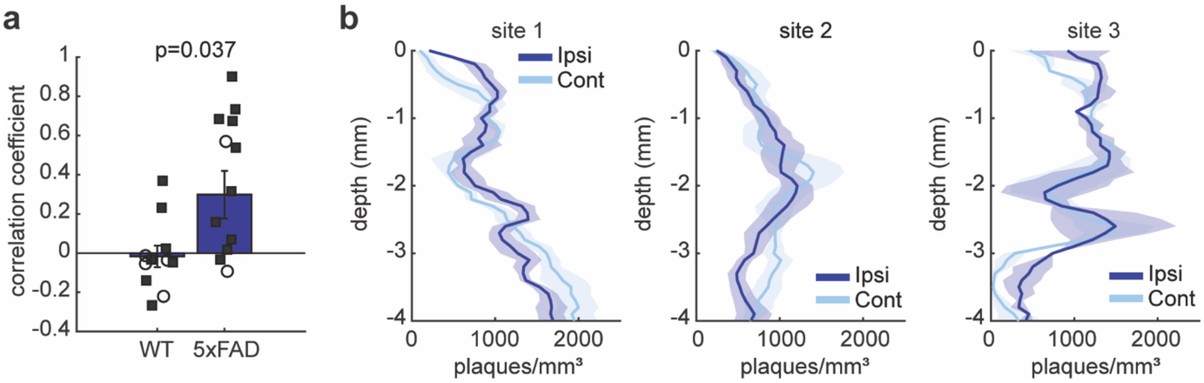

Supplemental Figure 4. Cross-hemisphere assessment of plaque density. (**a**) Summary correlation coefficients using quantified plaque density from the implanted (ipsilateral) hemisphere across three different implant sites (two-sample t-test). 5xFAD, n = 13 recordings from 6 mice. WT, n = 11 recordings from 7 mice. Squares, males; Circles, females. (**b**) Depth profile of plaque density across three different implant sites for the implanted (ipsilateral) and contralateral hemisphere.

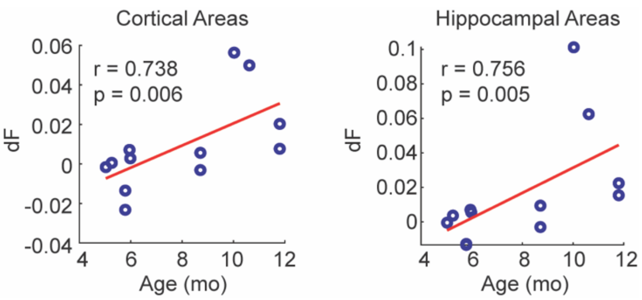

Supplemental Figure 5. Age-dependent increase in fluorescence in 5xFAD mice. Correlation between age and the *in vivo* fluorescent signals across cortical (*left*) and hippocampal (*right*) areas in 5xFAD mice (r, Spearman's Rho). Selected groups of galvo voltages corresponding to cortical and hippocampal regions determined the median *in vivo* photometry signals from 30-240 minutes for each region. n = 12 5xFAD mice.

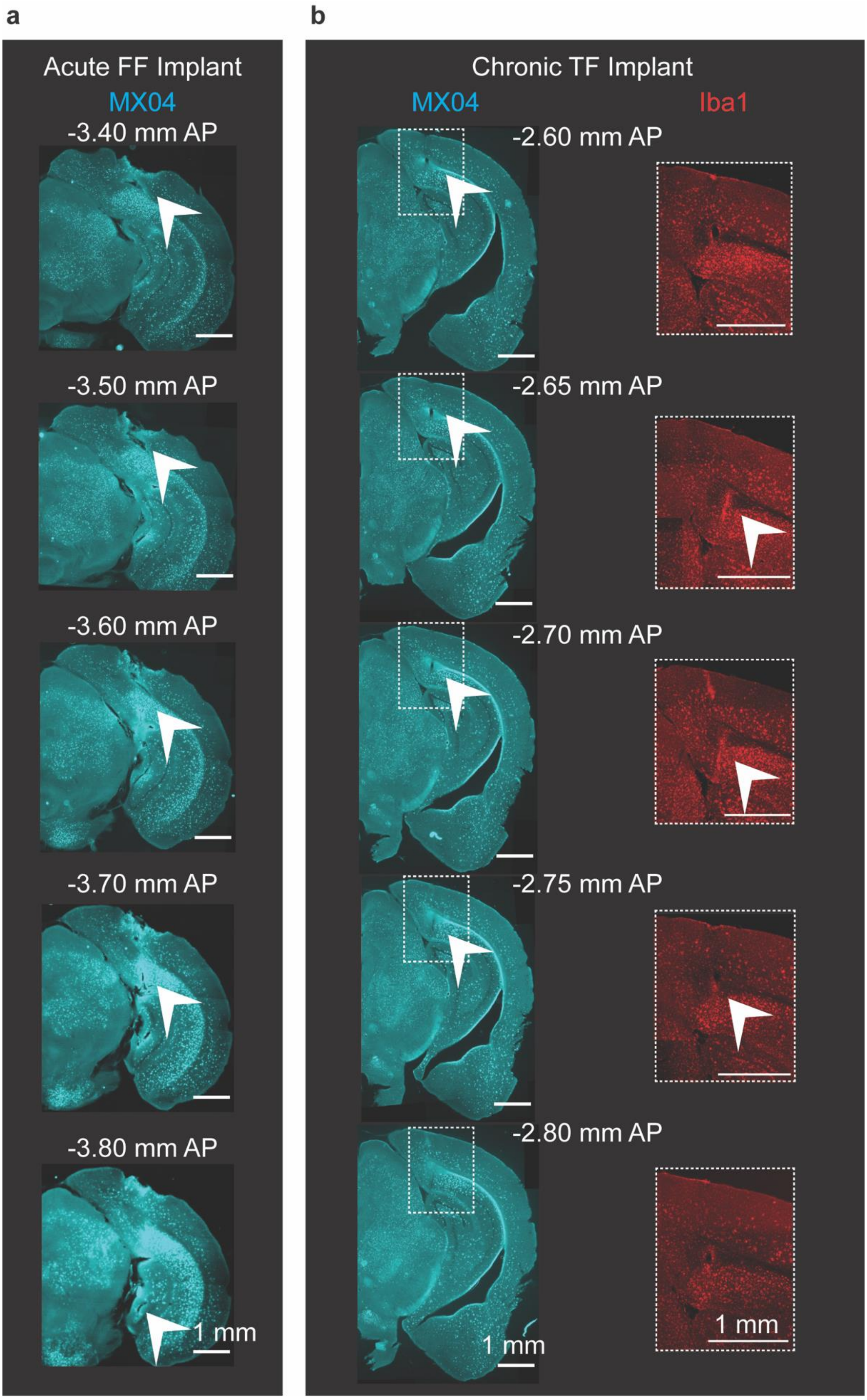

Supplemental Figure 6. Assessment of damage and microgliosis after acute FF and chronic TF implantation. (**a**) Coronal Methoxy-x04 stained sections across the AP axis show

tissue damage over a large area after an acute FF implant. White arrows show tissue damage. (**b**) Coronal Methoxy-x04 stained sections across the AP axis show less tissue damage and gliosis (zoomed inset in white dashed line) over a small area. White arrows show tissue damage and areas of microgliosis. Scale: 1 mm.
